# Exploring the association between serum vitamins and CAP-defined hepatic steatosis in lean and non-lean adults with NAFLD: A cross-sectional study

**DOI:** 10.1371/journal.pone.0346042

**Published:** 2026-04-13

**Authors:** Caijuan Hong, Lili Jiang, Lulu Xia

**Affiliations:** Hangzhou Linping District Integrated Traditional Chinese and Western Medicine Hospital, Hangzhou, Zhejiang, China; Sarich Neuroscience Research Institute, AUSTRALIA

## Abstract

**Background:**

Non-alcoholic fatty liver disease (NAFLD) is highly prevalent worldwide, yet the role of serum vitamins in hepatic fat accumulation, particularly across lean and non-lean individuals, remains incompletely understood. This study evaluated associations between serum levels of vitamins A, C, D, and E and the degree of CAP-defined hepatic steatosis in lean and non-lean adults.

**Methods:**

Data from the 2017–2018 NHANES were analyzed. Weighted multivariable linear regression was performed in lean and non-lean adults to assess the associations between serum vitamin levels and CAP-defined hepatic steatosis, using unadjusted, demographic-adjusted, and fully adjusted models.

**Results:**

No significant linear associations were observed between serum vitamin levels and the degree of hepatic steatosis in lean adults. A nonlinear association with serum vitamin C was observed, indicating a threshold effect. The association between vitamin C and the degree of liver fat accumulation also varied by diabetes status. In non-lean adults, serum vitamin C consistently showed an inverse association with CAP-defined hepatic steatosis (Model 2: β = −0.16, 95% CI: −0.25 to −0.07), as did vitamins A (β = −6.34, 95% CI: −10.43 to −2.25) and D (β = −0.13, 95% CI: −0.21 to −0.04).

**Conclusion:**

Higher serum levels of vitamins A, C, and D were associated with lower CAP-defined hepatic steatosis in non-lean adults, while a nonlinear relationship involving vitamin C was observed in lean individuals. Further studies are needed to confirm these findings.

## Introduction

Non-alcoholic fatty liver disease (NAFLD) is defined by the accumulation of lipids in the liver without the presence of excessive alcohol consumption or other liver disorders [1]. Over the past two decades, NAFLD has emerged as a leading cause of chronic liver disease, with its prevalence among U.S. adults estimated to be around 30% [2]. While hepatic steatosis is typically regarded as a benign condition, approximately 20–30% of affected individuals may progress to necroinflammation and liver fibrosis, a condition known as non-alcoholic steatohepatitis (NASH), which could ultimately lead to cirrhosis or hepatocellular carcinoma (HCC) [3,4]. A systematic review indicated that 40.8% of NAFLD cases are classified as non-obese fatty liver, while 19.2% fall under the category of lean fatty liver [5]. The mechanisms underlying liver fat accumulation in lean adults with hepatic steatosis may differ from those in their obese counterparts. Factors such as genetic predisposition, altered lipid metabolism, mitochondrial dysfunction, and insulin resistance independent of obesity have been implicated in the development of lean NAFLD [6]. Given these distinct pathogenic mechanisms, the influence of vitamins on lean versus non-lean adults with hepatic steatosis may also differ.

Vitamins are essential micronutrients that are vital for numerous biological functions. They significantly impact the immune system, affecting both innate and adaptive immune responses [7]. The immune system is intricately connected to the gut-liver axis and plays a crucial role in the initiation and progression of NAFLD. Recent research has emphasized the relationship between dietary vitamins and liver fat accumulation [8]. Patients with NAFLD frequently exhibit deficiencies in various vitamins, especially those with antioxidant properties, which has led to increasing clinical interest in the potential therapeutic use of antioxidant vitamins such as C, E, A, and D for treating NAFLD. Although recent investigations have focused on the effects of specific vitamins on liver metabolism, a comprehensive understanding of the broader role of vitamins in NAFLD remains limited [9], and the findings regarding the impact of these vitamins on NAFLD have been inconsistent.

Given the Inconsistent results from previous studies regarding the associations between vitamins A, C, D, and E and NAFLD, we conducted a cross-sectional study utilizing data from the 2017–2018 National Health and Nutrition Examination Survey (NHANES). This study aimed to evaluate the relationship between serum levels of vitamins A, C, D, and E and the degree of hepatic steatosis, as measured by controlled attenuation parameter (CAP). Additionally, we sought to determine whether the association between these vitamins and the degree of liver fat accumulation differs between lean and non-lean adults with CAP-defined hepatic steatosis.

## Materials and methods

### Study population

The National Health and Nutrition Examination Survey (NHANES) is a continuous cross-sectional study conducted in the United States, aimed at collecting a diverse array of multidimensional data. The study protocol received approval from the Research Ethics Review Board at the National Center for Health Statistics, and all participants or their guardians provided written informed consent during recruitment.

This analysis utilized data from the NHANES 2017–2018 cycle, which included a total of 9,254 participants. Within this cohort, 5,494 individuals underwent liver stiffness measurements. Participants aged under 20 years (n = 984) were excluded, leaving 4,510 adults for further screening.

Several exclusion criteria were then applied. Specifically, 43 individuals tested positive for HCV-RNA, while 259 had incomplete HCV data. Additionally, 27 participants were positive for hepatitis B surface antigen, and 18 had missing HBV data. Regarding alcohol consumption, 380 males who reported consuming more than three alcoholic beverages daily and 304 females who reported drinking over two beverages per day were excluded, resulting in 497 missing data points. In total, 2,448 participants were excluded based on these criteria.

**Fig 1:**
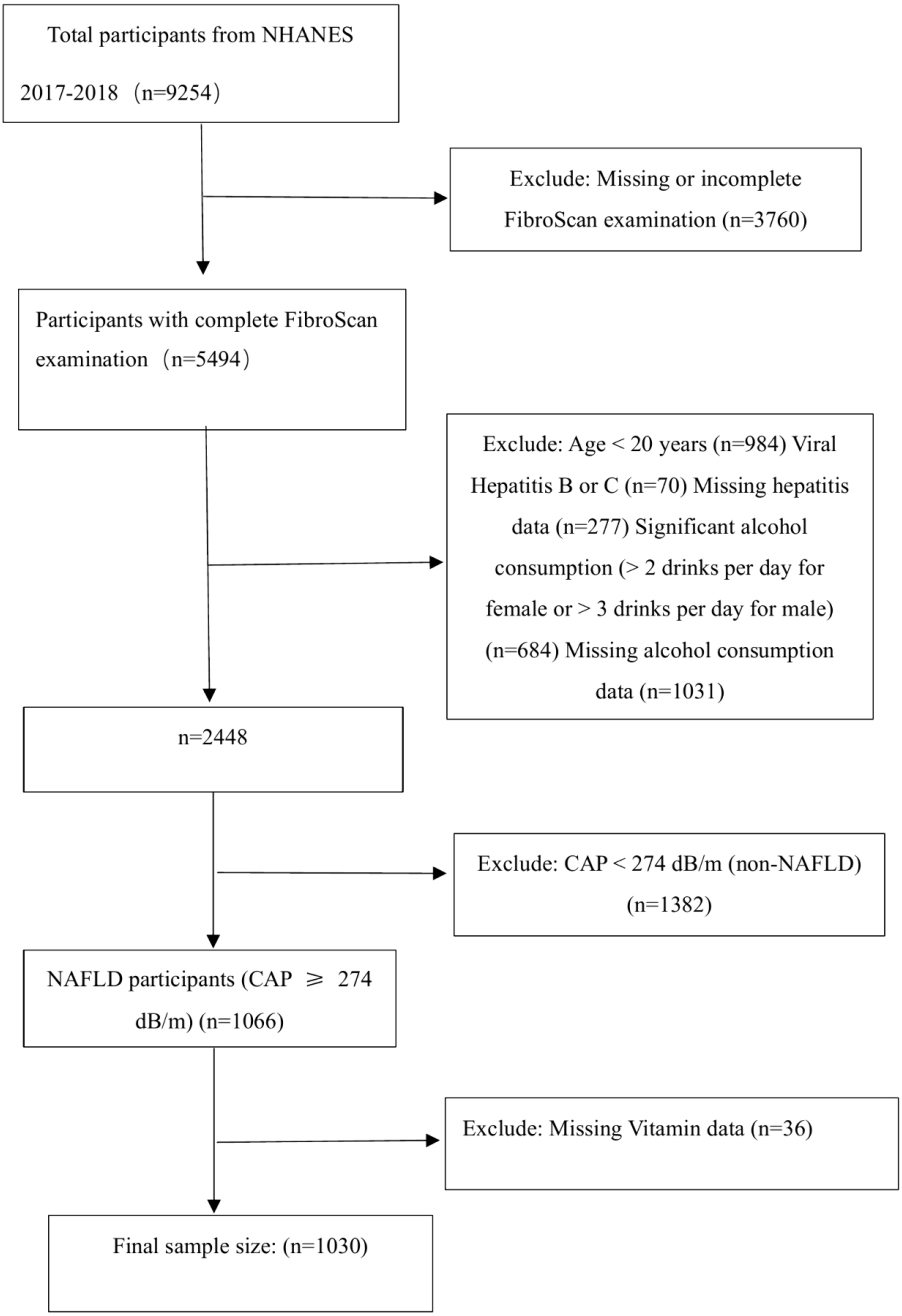
Flowchart of study participants selection.

Among the remaining individuals, 1,066 were identified as having hepatic steatosis, defined as a Controlled Attenuation Parameter (CAP) score ≥ 274 dB/m. After excluding 36 participants with missing vitamin data, the final study population consisted of 1,030 adults with hepatic steatosis (**Fig 1**).

Data were obtained from NHANES 2017–2018. Participants were excluded based on missing or incomplete FibroScan data, age < 20 years, viral hepatitis, and significant alcohol consumption. Complete transient elastography (FibroScan) examination was defined as a fasting time of ≥3 h, ≥ 10 complete liver stiffness measures, and a liver stiffness interquartile range/median <30%.

### Evaluation of hepatic steatosis

While liver biopsy remains the gold standard for evaluating hepatic steatosis, it is invasive, carries a risk of complications such as bleeding, is costly, and is not ideal for repeated evaluations. As a non-invasive alternative, the Controlled Attenuation Parameter (CAP), a novel technique based on ultrasound elastography, has gained wide use for quantifying liver fat content. In this study, liver steatosis was assessed using CAP obtained by vibration-controlled transient elastography (VCTE) (FibroScan®, Echosens, Paris, France). Studies have validated CAP against liver biopsy, demonstrating that its diagnostic performance in detecting hepatic steatosis is comparable to that of the gold standard [10]. A CAP threshold of ≥274 dB/m has been established as a criterion for diagnosing hepatic steatosis [11,12].

In the NHANES cohort, CAP measurements were taken under specific conditions: participants fasted for a minimum of three hours, obtained at least 10 valid liver stiffness (E) readings, and had an interquartile range to median liver stiffness ratio (IQRe/median) of less than 30%.

This method offers a dependable, non-invasive option for assessing the degree of hepatic steatosis, mitigating the risks and limitations of liver biopsy while preserving diagnostic precision.

### Variables

In this study, the exposure primary variables were the serum concentrations of vitamins A, C, D, and E. Serum retinol (vitamin A) and α-tocopherol (vitamin E) levels were measured using high-performance liquid chromatography with photodiode array detection (HPLC-PDA). Serum ascorbic acid (vitamin C) concentrations were measured via isocratic ultra-performance liquid chromatography (UPLC) with electrochemical detection at 450 mV. The total serum 25-hydroxyvitamin D [25(OH)D] concentration, defined as the sum of 25(OH)D2 and 25(OH)D3, was measured by the National Center for Environmental Health using high-performance liquid chromatography-tandem mass spectrometry (HPLC-MS/MS).

### Covariates

Several covariates were incorporated into the analysis to control for potential confounding factors, as highlighted in prior research. These included sex, age, race/ethnicity, poverty income ratio (PIR), body mass index (BMI), alcohol consumption (categorized as yes or no), smoking status (defined as having smoked at least 100 cigarettes during one’s lifetime), presence of diabetes (classified as yes, no, or prediabetes), hypertension (yes/no), and hypercholesterolemia (yes/no). These variables were chosen based on their established links with both serum vitamin concentrations and the degree of hepatic steatosis in comparable studies, ensuring that the analysis accounted for key demographic, socioeconomic, and metabolic influences on vitamin status and liver fat accumulation.

### Handling of missing data

In this study, participants with missing data for the primary exposure variables (serum vitamins A, C, D, and E) were excluded from the final analysis (listwise deletion). To minimize potential bias and maximize statistical power, missing values for covariates were imputed based on the distribution of each variable. Specifically, for continuous covariates exhibiting a normal or approximately normal distribution, missing values were replaced with the mean. For variables with skewed distributions, the median was used for imputation. This strategy allowed for the preservation of sample size and data integrity in the multivariable regression analyses.

### Statistical analysis

All analyses accounted for the complex survey design of NHANES using appropriate sample weights. Continuous variables were presented as weighted mean values with standard error (SE), and categorical variables were reported as weighted frequencies and percentages. Differences between groups were evaluated using weighted t-tests for continuous variables and weighted chi-square tests for categorical variables. Weighted multivariable linear regression models were employed to evaluate the association between serum vitamin concentrations and the degree of hepatic steatosis (continuous CAP score). Results were expressed as β coefficients with 95% confidence intervals (CIs). Three regression models were constructed: an unadjusted model; Model 1, adjusted for age, sex, and race/ethnicity; and Model 2, further adjusted for body mass index (BMI), poverty income ratio (PIR), smoking status, alcohol consumption, diabetes, hypertension, and hypercholesterolemia.

To evaluate potential non-linear relationships and threshold effects, smooth curve fitting (penalized spline method) and two-piecewise linear regression models were applied. A recursive algorithm was used to identify inflection points, and log-likelihood ratio tests were performed to compare the one-line linear model with the two-piecewise model. Subgroup analyses and interaction tests were conducted to investigate whether the associations varied by specific covariates. The significance of interactions was assessed using likelihood ratio tests.

All analyses were performed using R software (version 3.4.3, The R Foundation) and EmpowerStats (version 5.2, X&Y Solutions, Inc., Boston, MA), with a two-sided P-value < 0.05 considered statistically significant.

## Results

### Characteristics of the study population

The final analysis included 1,030 participants with CAP-defined hepatic steatosis, comprising 91 individuals with lean NAFLD and 939 with non-lean NAFLD, as classified according to predefined exclusion criteria (**Table 1**). The lean NAFLD group was significantly older on average compared to the non-lean group. Weighted analyses showed that serum concentrations of vitamins A, C, D, and E were significantly higher in the lean NAFLD group compared with the non-lean group (all P < 0.05). No statistically significant differences were observed between the two groups with respect to poverty income ratio (PIR), sex distribution, smoking status, or alcohol consumption. Similarly, the prevalence of diabetes, hypertension, and hypercholesterolemia did not differ significantly between the lean and non-lean groups.

**Table 1:** Characteristics of the study population.

Characteristics	Lean NAFLD (BMI < 25 kg/m²)	Non-lean NAFLD (BMI ≥ 25 kg/m²)	P-value
Total, n	91	939	
Age (years)	58.51 ± 14.71	53.56 ± 15.65	**0.004**
PIR	2.78 ± 1.61	2.82 ± 1.51	0.795
Vitamin A (µg/dL)	60.07 ± 16.36	55.21 ± 16.90	**0.009**
Vitamin C (mg/dL)	0.99 ± 0.51	0.86 ± 0.45	**0.007**
Vitamin D (nmol/L)	75.09 ± 31.07	66.77 ± 28.70	**0.009**
Vitamin E (µmol/L)	33.96 ± 11.60	31.00 ± 10.90	**0.014**
Sex, n (%)			0.852
Male	49 (53.85%)	496 (52.82%)	
Female	42 (46.15%)	443 (47.18%)	
Race/Ethnicity, n (%)			**<0.001**
Mexican American	6 (6.59%)	157 (16.72%)	
Other Hispanic	6 (6.59%)	83 (8.84%)	
Non-Hispanic White	30 (32.97%)	333 (35.46%)	
Non-Hispanic Black	8 (8.79%)	193 (20.55%)	
Other Race	41 (45.05%)	173 (18.42%)	
Smoking History, n (%)			0.802
≥100 cigarettes	33 (36.26%)	353 (37.59%)	
<100 cigarettes	58 (63.74%)	586 (62.41%)	
Alcohol Consumption, n (%)			0.125
Yes	71 (78.02%)	791 (84.24%)	
No	20 (21.98%)	148 (15.76%)	
Diabetes, n (%)			0.097
Diabetes	16 (17.58%)	214 (22.81%)	
No diabetes	67 (73.63%)	684 (72.92%)	
Prediabetes	8 (8.79%)	40 (4.26%)	
Hypercholesterolemia, n (%)			0.483
Yes	43 (47.78%)	409 (43.93%)	
No	47 (52.22%)	522 (56.07%)	
Hypertension, n (%)			0.068
Yes	35 (38.46%)	455 (48.46%)	
No	56 (61.54%)	484 (51.54%)	

Data are expressed as mean ± standard error (SE) for continuous variables and n (%) for categorical variables. P-values were calculated using weighted t-tests for continuous variables and weighted chi-square tests for categorical variables. Abbreviations: NAFLD, non-alcoholic fatty liver disease; BMI, body mass index; PIR, poverty income ratio.

### Associations between serum vitamins and hepatic steatosis

Weighted multivariable linear regression models were employed to evaluate the associations between serum vitamin levels and the degree of hepatic steatosis (CAP score) (**Table 2**).

**Table 2:** Weighted multivariable linear regression analysis of the association between serum vitamin levels and CAP-defined hepatic steatosis.

Exposure	Lean NAFLD (BMI < 25 kg/m²)	Non-Lean NAFLD (BMI ≥ 25 kg/m²)	Total
	β (95% CI) *P*-value	β (95% CI) *P*-value	β (95% CI) *P*-value
**Unadjusted**			
Vitamin A	0.61 (−8.92, 10.14) 0.8998	0.01 (−3.76, 3.78) 0.9951	0.06 (−3.48, 3.60) 0.9729
Vitamin C	−0.05 (−0.24, 0.13) 0.5823	**−0.17 (−0.26, −0.09) <0.0001**	**−0.16 (−0.24, −0.08) <0.0001**
Vitamin D	−0.08 (−0.26, 0.09) 0.3450	−0.03 (−0.11, 0.04) 0.4108	−0.04 (−0.11, 0.03) 0.3043
Vitamin E	0.17 (−0.29, 0.64) 0.4671	0.13 (−0.07, 0.34) 0.1996	0.14 (−0.05, 0.33) 0.1561
**Model 1**			
Vitamin A	2.51 (−7.45, 12.48) 0.6227	−3.77 (−7.79, 0.24) 0.0658	−3.31 (−7.07, 0.46) 0.0854
Vitamin C	0.02 (−0.17, 0.21) 0.8272	**−0.19 (−0.28, −0.10) <0.0001**	**−0.17 (−0.25, −0.09) <0.0001**
Vitamin D	−0.04 (−0.23, 0.14) 0.6699	**−0.11 (−0.20, −0.02) 0.0139**	**−0.10 (−0.18, −0.02) 0.0128**
Vitamin E	0.34 (−0.14, 0.82) 0.1699	0.08 (−0.13, 0.29) 0.4659	0.10 (−0.10, 0.30) 0.3144
**Model 2**			
Vitamin A	1.02 (−8.95, 10.99) 0.8421	**−6.34 (−10.43, −2.25) 0.0024**	**−5.90 (−9.73, −2.06) 0.0026**
Vitamin C	0.02 (−0.17, 0.21) 0.8169	**−0.16 (−0.25, −0.07) 0.0006**	**−0.14 (−0.23, −0.06) 0.0008**
Vitamin D	−0.13 (−0.33, 0.06) 0.1911	**−0.13 (−0.21, −0.04) 0.0045**	**−0.12 (−0.20, −0.04) 0.0029**
Vitamin E	0.12 (−0.42, 0.65) 0.6722	0.08 (−0.14, 0.29) 0.4818	0.08 (−0.12, 0.28) 0.4250

Abbreviations: CI, confidence interval; CAP, controlled attenuation parameter; BMI, body mass index; PIR, poverty income ratio.

Note: Data are presented as linear regression coefficients (β) and 95% CIs.

Model 1: Adjusted for age, sex, and race/ethnicity.

Model 2: Adjusted for age, sex, race/ethnicity, BMI, PIR, smoking status, alcohol consumption, diabetes, hypertension, and hypercholesterolemia.

Units for serum vitamins: Vitamin A (µmol/L), Vitamin C (µmol/L), Vitamin D (nmol/L), and Vitamin E (µmol/L).

In the non-lean NAFLD group, serum vitamin C demonstrated a robust inverse association with hepatic steatosis across all models. The β coefficient was −0.17 (95% CI: −0.26 to −0.09, P < 0.0001) in the unadjusted model, and this protective association persisted in the fully adjusted Model 2 (β = −0.16, 95% CI: −0.25 to −0.07, P = 0.0006). Similarly, in the fully adjusted Model 2, significant inverse associations were observed for serum vitamin A (β = −6.34, 95% CI: −10.43 to −2.25, P = 0.0024) and vitamin D (β = −0.13, 95% CI: −0.21 to −0.04, P = 0.0045), suggesting that higher levels of these vitamins were associated with lower CAP scores.

**Fig 2:**
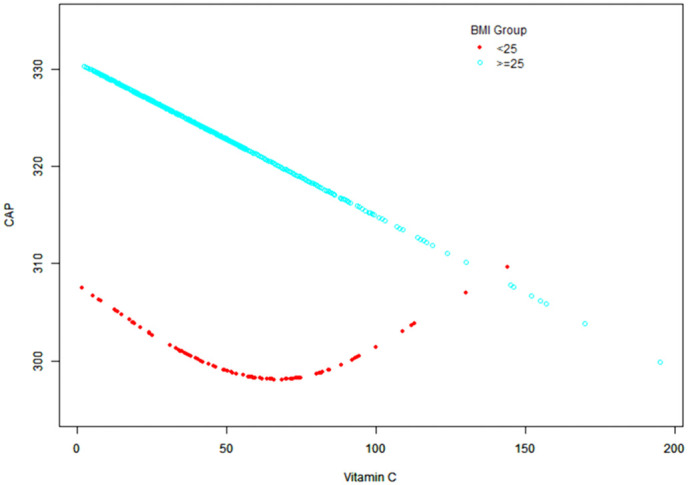
Smooth curve fitting analysis of the association between serum vitamin C levels and hepatic steatosis stratified by BMI.

In the lean NAFLD group, a non-linear relationship was observed between serum vitamin C and hepatic steatosis (**Fig 2**; **Table 3**). Threshold effect analysis identified an inflection point at 81.2 μmol/L. Below this threshold; higher vitamin C concentrations were associated with reduced hepatic steatosis. However, this protective effect was not evident at concentrations exceeding 81.2 μmol/L. No significant associations were found for vitamins A, D, or E in the lean group, nor for vitamin E in the non-lean group. Interaction analyses (**Table 4**) revealed a significant modifying effect of diabetes status on the relationship between vitamin C and hepatic steatosis specifically within the lean group (P for interaction = 0.021). In lean participants with diabetes, serum vitamin C levels were positively correlated with CAP scores (β = 0.44, 95% CI: 0.03, 0.84; P = 0.038), whereas no significant association was observed in no diabetes or prediabetes individuals. No significant interactions were observed regarding hypertension or hypercholesterolemia.

**Table 3:** Threshold effect analysis of serum vitamin C on hepatic steatosis using two-piecewise linear regression.

Outcome: CAP	Lean NAFLD (BMI < 25 kg/m²)	Non-Lean NAFLD (BMI ≥ 25 kg/m²)	Total
Model 1 (Linear)			
Linear effect	0.02 (−0.17, 0.21) 0.8169	**−0.16 (−0.25, −0.07) 0.0006**	**−0.14 (−0.23, −0.06) 0.0008**
*P* for interaction	0.336		
Model 2 (Non-linear)			
Inflection Point (K)	**81.2**	33.0	33.1
< K effect	−0.18 (−0.42, 0.07) 0.1599	−0.30 (−0.65, 0.05) 0.0970	**−0.34 (−0.67, −0.02) 0.0380**
> K effect	**0.64 (0.11, 1.16) 0.0201**	**−0.13 (−0.25, −0.01) 0.0381**	−0.10 (−0.21, 0.02) 0.0906
Difference	**0.81 (0.16, 1.46) 0.0168**	0.17 (−0.25, 0.59) 0.4266	0.25 (−0.14, 0.64) 0.2088
Predicted CAP	291.09 (281.70, 300.49)	323.87 (320.05, 327.70)	321.94 (318.29, 325.59)
Log-likelihood test	**0.008**	0.422	0.205
*P* for interaction	0.358		

Models were adjusted for age, sex, race/ethnicity, PIR, smoking status, alcohol consumption, diabetes, hypertension, and hypercholesterolemia. Data are presented as linear regression coefficients (β) and 95% confidence intervals (CIs). The unit for the serum vitamin C inflection point is µmol/L. Abbreviations: PIR, poverty income ratio; CI, confidence interval.

**Table 4:** Interaction analyses of serum vitamins and hepatic steatosis stratified by comorbidities (diabetes, hypertension, and hypercholesterolemia) in lean and non-lean NAFLD participants.

Stratification & Vitamins	Lean NAFLD (BMI < 25 kg/m²)			Non-lean NAFLD (BMI ≥ 25 kg/m²)		
	β (95% CI)	P value	P for interaction	β (95% CI)	P value	P for interaction
**Stratified by Diabetes Status**						
**Vitamin A**			0.132			0.215
No diabetes	3.08 (−7.46, 13.62)	0.568		−0.58 (−5.50, 4.34)	0.819	
Prediabetes	−47.81 (−103.53, 7.90)	0.096		−15.38 (−31.36, 0.60)	0.060	
Diabetes	−9.40 (−30.89, 12.10)	0.394		−2.75 (−9.10, 3.59)	0.395	
**Vitamin C**			**0.021**			0.778
No diabetes	−0.16 (−0.36, 0.05)	0.138		−0.18 (−0.28, −0.08)	<0.001	
Prediabetes	−0.48 (−1.32, 0.36)	0.262		−0.29 (−0.70, 0.13)	0.180	
Diabetes	0.44 (0.03, 0.84)	**0.038**		−0.13 (−0.31, 0.04)	0.139	
**Vitamin D**			0.172			0.154
No diabetes	−0.06 (−0.25, 0.12)	0.497		−0.04 (−0.14, 0.05)	0.366	
Prediabetes	−0.79 (−1.54, −0.04)	**0.043**		0.23 (−0.16, 0.62)	0.251	
Diabetes	−0.09 (−0.59, 0.41)	0.738		−0.15 (−0.29, −0.01)	**0.041**	
**Vitamin E**			0.087			0.200
No diabetes	−0.02 (−0.61, 0.56)	0.934		0.10 (−0.16, 0.36)	0.449	
Prediabetes	−0.97 (−2.33, 0.39)	0.166		0.97 (−0.16, 2.10)	0.092	
Diabetes	0.78 (−0.13, 1.69)	0.097		−0.08 (−0.42, 0.27)	0.658	
**Stratified by Hypertension**						
**Vitamin A**			0.335			0.421
No	5.86 (−8.46, 20.19)	0.425		0.18 (−5.78, 6.14)	0.953	
Yes	−3.61 (−17.05, 9.83)	0.600		−3.02 (−8.04, 2.01)	0.240	
**Vitamin C**			0.645			0.625
No	−0.02 (−0.26, 0.22)	0.879		−0.20 (−0.32, −0.07)	**0.002**	
Yes	−0.11 (−0.42, 0.20)	0.494		−0.15 (−0.27, −0.03)	**0.014**	
**Vitamin D**			0.761			0.602
No	−0.06 (−0.29, 0.16)	0.572		−0.04 (−0.16, 0.07)	0.463	
Yes	−0.12 (−0.42, 0.18)	0.433		−0.08 (−0.19, 0.02)	0.122	
**Vitamin E**			0.717			0.273
No	0.11 (−0.48, 0.70)	0.715		−0.02 (−0.30, 0.27)	0.900	
Yes	0.29 (−0.50, 1.08)	0.478		0.21 (−0.08, 0.50)	0.158	
**Stratified by Hypercholesterolemia**						
**Vitamin A**			0.659			0.619
No	0.61 (−15.06, 16.28)	0.940		0.07 (−5.64, 5.78)	0.980	
Yes	−3.86 (−16.67, 8.96)	0.557		−1.90 (−7.24, 3.43)	0.484	
**Vitamin C**			0.144			0.765
No	−0.24 (−0.51, 0.03)	0.081		−0.20 (−0.31, −0.08)	**0.001**	
Yes	0.04 (−0.23, 0.30)	0.794		−0.17 (−0.30, −0.04)	**0.010**	
**Vitamin D**			0.457			0.828
No	−0.25 (−0.52, 0.02)	0.074		−0.04 (−0.15, 0.07)	0.476	
Yes	−0.11 (−0.37, 0.14)	0.395		−0.06 (−0.17, 0.06)	0.349	
**Vitamin E**			0.164			0.791
No	−0.43 (−1.24, 0.38)	0.303		0.05 (−0.29, 0.40)	0.762	
Yes	0.30 (−0.36, 0.97)	0.375		0.11 (−0.16, 0.39)	0.422	

Models were adjusted for age, sex, race/ethnicity, PIR, smoking status, and alcohol consumption. Data are presented as linear regression coefficients (β) and 95% confidence intervals (CIs). Abbreviations: NAFLD, non-alcoholic fatty liver disease; PIR, poverty income ratio. Units for vitamins: Vitamin A (µmol/L), Vitamin C (µmol/L), Vitamin D (nmol/L), and Vitamin E (µmol/L). Bold values indicate statistical significance (P < 0.05). Sample sizes for each subgroup are detailed in Table 1.

The red dotted line represents lean NAFLD participants (BMI < 25 kg/m^2^), showing a non-linear relationship. The blue circles represent non-lean NAFLD participants (BMI ≥ 25 kg/m^2^), showing an inverse linear association. Models were adjusted for age, sex, race/ethnicity, PIR, smoking status, alcohol consumption, diabetes, hypertension, and hypercholesterolemia.

## Discussion

In this investigation, we evaluated the associations between serum concentrations of vitamins A, C, D, and E and the degree of hepatic steatosis (quantified by CAP) in both lean and non-lean phenotypes. Among participants with lean NAFLD, no significant linear associations were observed between serum vitamin levels and hepatic fat accumulation after multivariable adjustment.

In contrast, within the non-lean NAFLD group, serum vitamin C consistently demonstrated an inverse relationship with the degree of steatosis across all models. Additionally, serum vitamin A and vitamin D exhibited significant negative associations with hepatic fat in the fully adjusted models. No significant relationship was observed for vitamin E in either group.

### Micronutrient heterogeneity

Our findings highlight the specific roles of antioxidant vitamins in liver health, which may differ from those of other micronutrients. The complexity of these associations is illustrated by contrasting our results with a recent study by Jeong [13], which reported that dietary intakes of vitamins B1 and B3 (niacin) were not significantly associated with metabolic dysfunction-associated steatotic liver disease (MASLD), despite the theoretical role of niacin in lipid metabolism. This discrepancy suggests that vitamins acting primarily as antioxidants (A, C) or immunomodulators (D) may play a more direct role in mitigating the oxidative stress underlying hepatic steatosis compared to B-vitamins, which function predominantly as cofactors in energy metabolism. Furthermore, the same study identified low dairy product intake as a risk factor for MASLD. Given that dairy products are a primary dietary source of calcium and vitamin D, this finding aligns well with our observation that higher serum vitamin D levels are protective against hepatic steatosis.

### Vitamin A

Regarding vitamin A, previous studies have yielded inconsistent results. For instance, prior NHANES data (2007–2014) identified a negative association between vitamin A intake and NAFLD severity [14]. Conversely, other studies have reported positive correlations between serum retinol and NAFLD progression or fibrosis [15–17]. Prior research has established that vitamin A plays a pivotal role in regulating glucose and lipid metabolism in both hepatic and adipose tissues [18], and dysregulation of hepatic vitamin A homeostasis has been proposed as a contributing factor to NAFLD development [19]. These epidemiological inconsistencies likely stem from the biphasic nature of vitamin A metabolism. In early NAFLD, impaired hepatic storage may increase serum retinol, whereas advanced stages (fibrosis) lead to depletion of reserves and lower serum levels [20–22]. Additionally, the antioxidant effects of vitamin A may be modulated by obesity-induced inflammation [23], potentially explaining the lack of association in our lean subgroup.

### Vitamin C

Vitamin C deficiency has been linked to dyslipidemia and hepatic oxidative stress, while supplementation has shown hepatoprotective effects in animal models [24,25]. Consistent with prior US-based studies [26], we found a strong inverse relationship between serum vitamin C and steatosis in non-lean individuals. Notably, we observed a novel nonlinear “threshold effect” in the lean NAFLD group. Below a serum concentration of 81.2 μmol/L, vitamin C appeared protective; however, above this threshold, a positive correlation with steatosis was observed. This paradoxical trend suggests that at distinctively high physiological concentrations, or in the specific metabolic context of lean NAFLD, vitamin C might exhibit pro-oxidant properties or interact with other metabolic pathways. Furthermore, our interaction analysis revealed that this relationship is significantly modified by diabetes status, suggesting that the metabolic derangements associated with diabetes may alter the utilization or impact of vitamin C in lean individuals.

### Vitamin D and E

Vitamin D is an essential steroid hormone implicated in the pathogenesis of chronic liver diseases [27], and its deficiency is widely recognized as a risk factor for NAFLD severity [28]. However, prior studies specifically using CAP have yielded inconsistent results regarding its association with hepatic steatosis [29,30], although inverse associations with fibrosis have been noted [31]. Our findings of a negative association in the non-lean group add to this evidence, supporting the hypothesis that vitamin D exerts anti-inflammatory effects. Regarding vitamin E, it is a well-established potent lipophilic antioxidant [32]. Meta-analyses of randomized controlled trials have demonstrated that vitamin E supplementation can improve liver histology and biochemistry in NASH patients [33,34]. In contrast, we did not observe a significant association between serum vitamin E levels and hepatic steatosis. This discrepancy aligns with other population-based observational studies [35] and likely reflects the difference between physiological serum levels and the high pharmacological doses used in intervention trials.

### Strengths and limitations

This study has several strengths, including the use of a nationally representative sample, the stratification by BMI phenotype, and the exploration of non-linear relationships. However, limitations must be acknowledged. First, the cross-sectional design precludes causal inference; reverse causality cannot be excluded, as liver disease itself may alter vitamin metabolism. Second, while we adjusted for BMI and macronutrients, we did not adjust for total energy intake derived from dietary recall due to potential measurement bias, which remains a residual confounder. Third, the sample size of the lean NAFLD group (n = 91) was relatively small, which may limit statistical power and generalizability. Finally, serum concentrations reflect internal exposure but do not perfectly differentiate between dietary intake, absorption efficiency, and tissue storage. Future longitudinal studies are warranted to validate these findings and elucidate the mechanistic pathways.

## Conclusion

This study indicates that serum concentrations of vitamins A, C, and D are inversely associated with the degree of hepatic steatosis, as quantified by CAP, among individuals with non-lean NAFLD, whereas no significant associations were observed for vitamins A and D in the lean NAFLD group. In the lean NAFLD group, a non-linear association between serum vitamin C and hepatic steatosis was observed, with an inverse relationship below an inflection point of 81.2 μmol/L and no evidence of a protective effect beyond this threshold. No significant associations were observed between serum vitamin E concentrations and hepatic steatosis in either group. These findings highlight the need for larger, prospective studies to better clarify these relationships and establish causality. The consistent inverse associations of vitamins A, C, and D with steatosis in non-lean NAFLD suggest potential therapeutic value that warrants further investigation.

Collectively, these findings underscore the metabolic heterogeneity between lean and non-lean NAFLD phenotypes. Larger, well-designed prospective studies are warranted to validate the observed dose–response relationships, clarify temporal associations, and elucidate the underlying mechanisms linking micronutrient metabolism to hepatic steatosis. The consistent inverse associations of vitamins A, C, and D with hepatic steatosis in non-lean NAFLD suggest a potential role as biomarkers or modifiable nutritional factors, meriting further investigation.

## Supporting information

S1 Data Analytic dataset used in this study. (XLSX)
